# Why We Should Study Multimodal Language

**DOI:** 10.3389/fpsyg.2018.01109

**Published:** 2018-06-28

**Authors:** Pamela Perniss

**Affiliations:** School of Humanities, University of Brighton, Brighton, United Kingdom

**Keywords:** multimodality, language, communication, sign language, gesture

What do we study when we study language? Our theories of language, and particularly our theories of the cognitive and neural underpinnings of language, have developed primarily from the investigation of spoken language. Moreover, spoken language has been studied primarily as a unichannel phenomenon, i.e., as just speech or text. However, contexts of face-to-face interaction form the primary ecological niche of language, both spoken and signed, and the primary contexts in which language is used, is learned and has evolved (Levinson and Enfield, 2006; Vigliocco et al., 2014). In such contexts, a multitude of cues, both vocal and visual, contribute to utterance construction. Should we thus not turn our attention to the study of language as multimodal language? The position that language can be appropriately studied as just speech or text essentially aligns with a conception of language based on Chomsky's *competence* or Saussure's *langue*: it is the linguistic code and the formalization of phonological, morphological, and syntactic structure that is of interest. Even functional, usage-based theories of language, which see linguistic structure as shaped by language use and the function of language in cultural and communicative contexts (e.g., Fillmore, 1982; Givón, 1984; Goldberg, 1995), have focused on the linguistic code and have thus also mainly regarded language as speech or text (but see e.g., Tomasello, 1999; Diessel, 2006). The argument put forward here is that we should study language as its multimodal manifestation in contexts of face-to-face interaction. As such, our object of study should subsume information expressed in both vocal and visual channels, including prosody, gesture, facial expression, body movement, which invariably accompany linguistic expression in face-to-face contexts.

The thought experiment proposed by Vigliocco et al. (2014) offers a window onto this approach by asking: *What if the study of language had started with the study of signed language rather than spoken language?* If the study of language had started with signed language, the multichannel/multimodal nature of language would have stood center stage from the beginning. Questions that have become matters of serious inquiry and debate only recently, in particular concerning the status and interplay of iconicity and arbitrariness (Perniss et al., 2010; Perniss and Vigliocco, 2014; Dingemanse et al., 2015) and gradience and categoricity (see Goldin-Meadow and Brentari, 2017 and peer commentary, e.g., Occhino and Wilcox, 2017, for review) in language, may have been discussed earlier and answered in different ways. This brings to the fore the relevance of thinking about language in a more unified way: encompassing spoken and signed language; considering multiple channels of expression; and conceptualizing language with respect to its communicative functions.

What have been considered to be non-linguistic aspects of communication—including gesture, facial expression, body movement—have largely been studied separately from language proper. Multimodality studies, for example, are often framed as offering analyses of social interaction, studying something that is around language, but not studying language as such (see Mondada, 2016 for an overview). Pioneering scholars in the field of gesture studies have long advocated for a conception of gesture that is part and parcel of language (McNeill, 1985, 1992; Kendon, 2004). Nevertheless, this conception has not been adopted on a large scale. In advocating for a multimodal conception of “language,” it is important to bear in mind the extent to which our objects of study are constructed by an interplay of the present state of theory, technology and discourse (Kuhn, 1962; Foucault, 1972). This point is made by McNeill (1985: 350) when he writes that the division between speech and gesture (or “body language”) is “a cultural artifact, an arbitrary limitation derived from a particular historical evolution”—they are studied separately, though McNeill considers them to be “parts of a single psychological structure.” The conception that “language” is that which is linguistic, while communication is something different—essentially, the Saussurean and Chomskyan heritage—is not given by necessity. As such, it is time to reconceptualize our object of study and to usher in a new paradigm of language theory, a paradigm that focuses on multimodal language, that aligns with the real world use of language and focuses on *doing language* (Andresen, 2014; Kendon, 2014).

The study of sign language and gesture, as communicative expression in the visual modality, has been instrumental in widening the lens of investigation regarding the question of our object of study when we study language. Signed language highlights the fundamental multimodality and semiotic diversity of language. Moreover, the study of sign language, and its comparisons with speech and/or gesture, has highlighted the difficulties of maintaining a principled distinction between the linguistic and non-linguistic, and shown the need for developing analyses that admit a combination of categorical (considered linguistic) and gradient (considered non-linguistic) aspects of language (Liddell, 2003; Johnston, 2013; Kendon, 2014; Vigliocco et al., 2014; Goldin-Meadow and Brentari, 2017). Similarly, gesture and multimodality research has shown that, like signers, speakers make use of a wide range of semiotic resources, combining vocal and visible action in meaning making and utterance construction (e.g., Kendon, 2004; Mondada, 2016). The study of sign and gesture expose our current models of language as too narrowly conceived. The new paradigm for the study of language must acknowledge a range of semiotic practices (exhibiting iconicity, arbitrariness, gradience, categoricity) as fundamental to and constitutive of communicative expression. Below, I outline developments in contemporary research that further attest to the need for incorporating multimodality into our theories of language.

The neuroscientific investigation of language processing is one area in which the distinction between “language” and “communication,” and between “linguistic” and “non-linguistic” elements has been undermined. Recent research has been unable to find strong evidence supporting this distinction in language use. In addition, there is evidence that the brain does not privilege linguistic information in processing. Rather all kinds of context, including multimodal cues, are processed simultaneously and immediately (Hagoort and van Berkum, 2007). Numerous studies have provided evidence for similar processing of gesture and speech in terms of semantic and temporal integration (Özyürek et al., 2007; Hubbard et al., 2009; Straube et al., 2009; Habets et al., 2011; Dick et al., 2014; Yang et al., 2015; Peeters et al., 2017), as well as in terms of perceiving conventionalized meaning (Andric et al., 2013; Wolf et al., 2017). In addition, there is evidence that prosodic information from visual and vocal channels is treated similarly by the brain, with gestural beats functioning as visual prosody complementary to speech prosody (Biau et al., 2016). Studies also suggest that the use of different cues from context, including co-speech gesture (Skipper, 2014; Weisberg et al., 2017) and visible mouth movements (van Wassenhove et al., 2005), may speed up processing, aiding interpretation through improved prediction, and requiring less allocation of neural resources and thus conserving metabolic resources. Similar processing of semantically meaningful information, regardless of the modality of presentation has, crucially, also been shown for processing of signed and spoken language (MacSweeney et al., 2004) as well as for integration of pictures with sentence context (Willems et al., 2008). Thus, recent evidence from neuroimaging studies does not support a principled divide between linguistic and non-linguistic elements as the legacy of studying language as *competence* or *langue* presupposes. Instead, the evidence suggests that the brain is specially attuned to *doing language* or *languaging* (Andresen, 2014; Kendon, 2014).

Additional evidence supporting a multimodal view of language comes from recent research that suggests that what has traditionally been considered to be non-linguistic may in fact be subsumable under grammar and susceptible of grammatical description. Floyd (2016), describing the obligatory incorporation of celestial pointing gestures for time-of-day reference, discusses the possibility of modality hybrid grammars, which would incorporate gestural forms into the grammar. Recent work by Schlenker and Chemla (2017), aims to provide evidence for the grammar-like nature of gestures. Similarly, Ginzburg and Poesio (2016) offer a formalization of intrinsically interactional aspects of language, including gestures as well as disfluencies and non-sentential utterances, with the goal of demonstrating their grammatical, rule-governed behavior. This resonates with work by gesture researchers who have sought to define multimodal approaches to grammar (e.g., Mittelberg, 2006; Fricke, 2012), and who have studied aspects of conventionality in gesture, identifying varying degrees of conventionality in form-meaning pairings in gesture, used consistently across speakers within language communities for conveying certain meanings (e.g., Kendon, 1995, 2004; Calbris, 2011; Bressem and Müller, 2017; Bressem et al., 2017; Müller, 2017). Similarly, elements in the vocal modality not traditionally considered to be linguistic have been found to exhibit systematic behavior in terms of discursive and interactional function, e.g., research on the use of clicks and percussives (Wright, 2011; Ogden, 2013) and “filled pauses” like *uh* and *um* (Clark and Fox Tree, 2002).

Technological advances in experimental paradigms, data collection and analysis further motivate the need for a new paradigm in the study of language. The need for experimental control has meant that ecological validity, and the study of language in more real-world settings, has often been sacrificed (Hasson and Honey, 2012). Experimental limitations in the past have thus constrained researchers to the study of certain aspects of language. These aspects have happened to align with a *langue/competence*-type object of study, best represented as individual words (spoken or written lexemes) and combinations of words (spoken or written sentences). “Non-linguistic” elements, e.g., gradient and iconic elements which naturally occur in parallel and simultaneously with the abstractable, formal linguistic elements, were excluded from study (Tromp et al., 2017). In addition, the wider so-called extra-linguistic context, given by the environment—full of visual and acoustic cues—in which language typically occurs was likewise excluded from study (Knoeferle, 2015). However, new methodologies, and in particular, combinations of methodologies (e.g., Virtual Reality environments with ERP, Tromp et al., 2017; eye-tracking with ERP, Knoeferle, 2015) can improve the interpretation of data from a single methodology. Overall, the development of these technologies will support the construction of multimodal language (in the active sense of *doing language*) as the new object of study, which more resembles real-world use of language, rather than being restricted to just one aspect of it (Kendon, 2009). These technologies will allow investigation of the use and processing of language in more ecologically valid, contextually rich and communicatively real-world settings.

Renewed interest in the evolutionary origins of language also points toward a focus on the multimodality of language. One question that has dominated the discourse on theories of language evolution concerns the modality of early communication. Adherents to the “gesture-first” theory of language (e.g., Corballis, 2002, 2017; Tomasello, 2008; Arbib, 2012) claim that symbolic communication originated in the visual-manual modality, and that there was, over time, a transition to the vocal channel as the main carrier of linguistic function. However, eminent gesture researchers like McNeill (1992, 2012) and Kendon (2009, 2017) have claimed that expression in the vocal and visual modalities must have characterized communication from the very start (see also Perlman, 2017). The explanation of a “switch” from the visual to the vocal modality is difficult to motivate, and the tight semantic and temporal orchestration of multiple channels of expression and semiotic resources observable today (from corpus to neuroimaging studies) suggests that utterance construction has always shown this entanglement of modes. In addition, the evidence supporting tight hand-mouth coordination and links between kinesis (e.g., grip) and vocalization (Gentilucci et al., 2001; Kendon, 2009; Vainio et al., 2013) further support a view that gives the “speech-kinesis ensemble” (Kendon, 2009) pride of place in the phylogenetic evolution of language. Interesting perspectives for the interplay of visual and vocal communication supporting language emergence *ab initio* comes from comparative psychology and animal cognition (Leavens, 2003; Gillespie-Lynch et al., 2014) and from the suggestion by Larsson (2015) that the sounds of tool use and locomotion may have contributed to language evolution in a similar way as visible action and motion. Taking “multimodal language” as our object of study would allow a straightforward reconciliation of such findings.

Finally, developments in the fields of multilingualism research and language documentation offer illustrative guides to the changes that need to be generalized in language theory more broadly. The field of multilingualism research has recently been transformed through the notion of *translanguaging*. Researchers no longer conceive of *code-switching* or even *code-mixing* as an adequate account of the language behavior of bi-/multilingual speakers (Li, 2017). Bi-/multilingual speakers do not switch between or mix different “codes,” as formal systems of language. Rather, they engage in flexible use of diverse semiotic repertoires. Kusters et al. (2017) note that in translanguaging studies, researchers focus on multilingual communication, but without paying attention to multimodal communicative resources; while in multimodality studies, researchers do not attend to multilingual communication. Given the parallels with respect to the focus on a diverse semiotic repertoire and dynamic language practice, Kusters et al. (2017) note the benefits of bringing the fields together, and suggest that the language practices of signers can offer unique insight into the use and negotiation of both multimodal and multilingual repertoires.

Many linguists, especially those studying endangered languages, have adopted practices consistent with the linguistic subdiscipline of language documentation (Himmelmann, 2006). The goal of language documentation goes beyond the production of a (written) grammar of a language. Rather, the goal is documentation of language use and practice in order to create a “lasting, multipurpose record of a language” (Himmelmann, 2006, p. 1). Technological advances have been a boon here as well. Language documentation demands video-recordings of language use on as broad a scale as possible, including different varieties of use, domains of use, and social interaction. This necessarily includes the multimodality of language, and attention to multichannel and semiotically diverse modes of communication. The recognition that the majority of the world is multilingual is also important here, in that it points to the inadequacy of characterizing knowledge of language as residing in an idealized, monolingual speaker in a homogenous language community (Chomsky, 1965). Ansaldo (2010, p. 622) suggests that lessons from monolingual language use and transmission may represent such “exotic communicative ecologies in the history of human language evolution [that] the lessons derived from their study, albeit significant, could well end up being potentially exceptional, maybe even peripheral to the construction of general theories of language.”

Similarly, our models of language need to be based on ecologically valid contexts of multimodal language use (contexts of *doing language*)—and not on the “exotic communicative ecologies” represented by just speech or text. The development of our hitherto dominant models of language has been based on only a part of language, the abstractable, linguistic part best exemplified by written form (McNeill, 1985). A multimodal language model includes the full complement of fundamental modes of communication, including depiction, description, and indexing (Clark, 1996, 2016), and the wider context in which utterances are constructed and interpreted (Kendon, 2014; Vigliocco et al., 2014; Knoeferle, 2015). In various and interconnected ways, the studies reviewed above suggest that we are already on the threshold of a new paradigm. They point to the large range of elements, both vocal and visual, that contribute in systematic ways to language use and communicative expression and which we should not exclude a priori from the study of language (See Andrén (2014) for discussion of the nature of the problem of delineating the “lower limit of gesture”—the problem of drawing a line between what aspects of “visible action as utterance” Kendon (2004) to include or exclude from study.). We must remind ourselves that science often progresses precisely through a redefinition of the object of study. By redefining the nature and parameters of our concept of “language” we will be capable of forging this new paradigm adequate to a unified conception of language as communication, and basing our theories of language on language as a multimodal phenomenon.

## Author contributions

The author confirms being the sole contributor of this work and approved it for publication.

### Conflict of interest statement

The author declares that the research was conducted in the absence of any commercial or financial relationships that could be construed as a potential conflict of interest.
